# A Smartphone Serious Game for Adolescents (Grow It! App): Development, Feasibility, and Acceptance Study

**DOI:** 10.2196/29832

**Published:** 2022-03-03

**Authors:** Evelien Dietvorst, Michelle A Aukes, Jeroen S Legerstee, Annabel Vreeker, Micah M Hrehovcsik, Loes Keijsers, Manon H J Hillegers

**Affiliations:** 1 Department of Child and Adolescent Psychiatry/Psychology Erasmus MC Sophia Children’s Hospital Erasmus University Medical Center Rotterdam Netherlands; 2 Innovation Studio HKU University of the Arts Utrecht Utrecht Netherlands; 3 Erasmus School of Social and Behavioural Sciences Department of Psychology Education & Child Studies Rotterdam Netherlands

**Keywords:** ecological momentary assessment, EMA, serious game, CBT, depression, internalizing problems, adolescents, high risk, digital health, mobile health, mHealth, game design, app development, mobile phone

## Abstract

**Background:**

Anxiety and mood problems in adolescents often go unnoticed and may therefore remain untreated. Identifying and preventing the development of emotional problems requires monitoring and effective tools to strengthen adolescents' resilience, for example, by enhancing coping skills.

**Objective:**

This study describes the developmental process, feasibility, and acceptance of Grow It!, a multiplayer serious game app for adolescents aged 12-25 years. The app consists of the experience sampling method (ESM) to monitor thoughts, behaviors, and emotions in daily life to enhance self-insight and daily cognitive behavioral therapy–based challenges to promote adaptive coping.

**Methods:**

Our approach entails an iterative game design process combined with an agile method to develop the smartphone app. The incorporated game features (ie, challenges, chat functionality, and visual representation) in the Grow It! app were co-designed with adolescent end users to increase participant engagement and adherence.

**Results:**

The Grow It! app was delivered for Android and iOS in May 2020. Grow It! was offered to adolescents during the COVID-19 crisis between May and December 2020. Participants of the Grow It! COVID-19 study (sample 1: N=685; mean age 16.19, SD 3.11 years; 193/685, 28.2% boys; sample 2: N=1035; mean age 18.78, SD 3.51 years; 193/1035, 18.64% boys) completed 31.5% (13.2/42) to 49.5% (10.4/21) of challenges. Compliance of ESM was suboptimal (35.1/210, 16.7% to 32.5/105, 30.9%). Follow-up questionnaires indicated an overall score of the app of 7.1 out of 10. Moreover, 72.6% (278/383) to 75.6% (487/644) would recommend the app to friends.

**Conclusions:**

To our knowledge, Grow It! is the first gamified ESM app that both measures individual differences in emotional dynamics and offers an integrated cognitive behavioral therapy–based intervention. Our findings support the feasibility and acceptance, and therefore applicability, of the Grow It! app in adolescents. Further iterations of this serious game app will focus on the increase of compliance and on providing participants feedback through their personal mood profiles.

## Introduction

### Background

Internalizing problems, such as anxiety and mood problems, have a substantial impact on young people’s lives. These internalizing problems are often associated with school dropout, reduced social functioning, loneliness, unemployment, and reduced quality of life [1-3]. Anxiety and mood disorders usually begin in adolescence [4,5], and unfortunately, they often go unnoticed or untreated [6]. When persistent, internalizing problems often result in emerging psychiatric disorders affecting young people’s daily lives, their future, and society [1-3,6]. Therefore, early identification and timely intervention are crucial to prevent further deterioration, improve prognosis, and reduce the burden on health care systems and society in general [1,7,8].

Mobile health (mHealth) can play an important role in accurate recognition of symptoms and timely treatment [9-11]. mHealth is defined as wireless technologies, such as smartphone apps, to support or achieve health objectives. In terms of its advantages, first of all, mHealth is scalable, accessible, and maybe less stigmatizing than traditional treatment for youths because of the level of anonymity and privacy [12]. Furthermore, mHealth offers the possibility of incorporating motivational elements such as playfulness and gamification, which is advantageous because humans supposedly learn best by playing [13-17]. Finally, mHealth offered in an attractive and fun way through adolescents’ own devices fits very well with their daily life and activities [18,19]. Use is flexible, as it is independent of time and place and can be at a self-determined pace, which is thought to enhance self-efficacy [12]. Indeed, most adolescents indicated that they would use an app to screen for emotional problems and treatment if available [20]. Even though there are already several mHealth apps [21], preventive mHealth apps that integrate novel methods for early identification and preventive intervention are still lacking.

A promising method for the early identification of emotional problems is the experience sampling method (ESM). The ESM is a structured diary method in which participants obtain multiple random notifications on their phone during the day. When a notification pops up, they fill out a microquestionnaire in which they report on their behaviors, thoughts, and feelings in real time (eg, how are you doing right now?). The strength of the ESM is the high ecological validity. Moment-to-moment assessments in real-world settings address the problem of recall bias [22,23]. The promise of ESM data for early identification of adolescents at risk for the development of psychopathology has been demonstrated in a research setting with adults [24] and adolescents [25]. Moreover, self-management may be enhanced by obtaining insights into everyday functioning dynamics, based on ESM data [22,26,27].

The first choice of treatment of anxiety and mood problems in adolescents is psychological therapy, especially cognitive behavioral therapy (CBT) [28-30]. It is known that the way adolescents cope with stress or handle negative emotions in daily life may increase or buffer against the development of anxiety and depressive symptoms [31-33]. That is why CBT is one of the effective interventions aimed at improving coping. Recently, results of an internet-based CBT intervention revealed that positive effects occur already after 4 weeks of CBT [34]. CBT is mostly used in clinical practice but increasingly also applied for preventive purposes [28,29].

Therefore, we cocreated the Grow It! app (Android and iOS) for adolescents aged 12 to 25 years. Grow It! is a multiplayer serious game, a game that is designed for a primary purpose other than entertainment [30]. Incorporated in the Grow It! app are ESM, to enhance self-management and identify mood problems earlier on, and gamified CBT-based challenges, to increase coping. Initially, the app was developed for high-risk adolescent populations, such as adolescents with chronic somatic conditions, offspring of parents with psychiatric disorders, or adolescents experiencing extreme stressful societal circumstances, for example, the COVID-19 pandemic. However, the app may also serve a broader purpose of prevention for adolescents from a general population.

### Objectives

The first aim of this study is to give an elaborate description of the developmental process of the multiplayer serious game app Grow It! Second, we aim to study the feasibility and acceptance of Grow It! among end users.

## Methods

### Developmental Process

#### Overview

**Figure 1:**
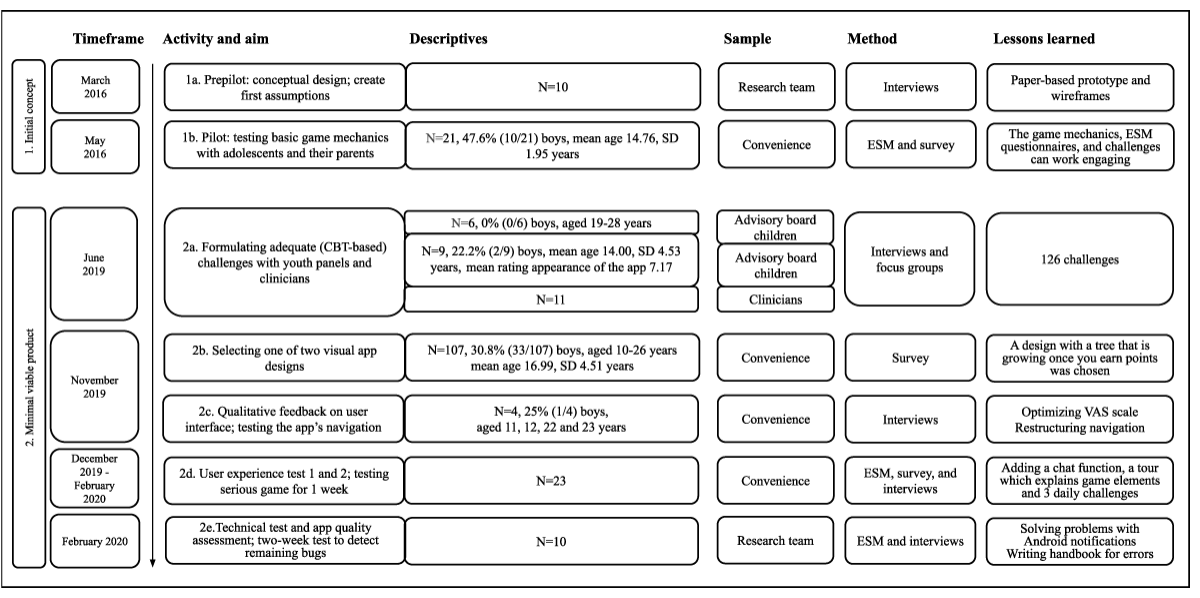
The developmental process of the Grow It! app. ESM: experience sampling method; VAS: visual analog scale.

The Grow It! app was developed, with intermittent periods, from March 2016 to February 2020 (Figure 1). During its development, we cocreated the app with a large multidisciplinary team of child and adolescent psychiatrists, developmental and clinical psychologists, data analysts, game designers, and multiple test panels (adolescents aged 12-25 years) [35,36]. The initial concept was developed with University of the Arts Utrecht (test 1a-1b). With IJsfontein BV and consultancy company Game Architect Studio, an agile process (defined as a software development methodology including iterative development, where requirements and solutions evolve in multidisciplinary teams [37]) then was used to develop and evaluate the minimal viable product (MVP; test 2a-2e). For all tests, informed consent was obtained.

#### Initial Concept

In March 2016, the developmental process of Grow It! started with a prepilot (test 1a, N=10), which resulted in a paper-based prototype and wireframes. Later on, in the pilot (test 1b), adolescents from the general population (N=21) tested the app’s beta version during a 6-week trial. Weeks 1 and 6 of the test consisted of an ESM-only study with 8 mood assessments per day. During weeks 2 to 5, the mood assessments were given twice a day and combined with CBT-based challenges. In all weeks, users received feedback through push messages complimenting them. Lessons learned were that adolescents were motivated by the game mechanics of Grow It! and liked completing the ESM questionnaires and daily challenges.

#### Minimal Viable Product

In June 2019, an MVP was built. We aimed to improve the app's content, visual design, interaction design, and reliability of assessments and ran a technical test. The app was developed using agile development and user-centered design methods, including different tests and collaboration with focus groups. Different groups of adolescents (n=6 and n=9) received instructions and were invited to design CBT-based challenges aiming at adaptive coping [36]. Thereafter, all ideas were formulated into specific challenges and were rated in terms of their clinical appropriateness and coping effectiveness by 11 child and youth psychologists and psychiatrists. As a result of these focus groups (test 2a), 126 challenges were formulated, which were later used as the challenges in the Grow It! app. Furthermore, a survey among 107 adolescents (test 2b) resulted in the choice for a visual design that (1) was accepted by a broad range of ages and by both boys and girls and (2) was low cost in maintenance. In interviews (N=4, test 2c) with regard to the navigation of the app, special attention was given to the answer-scale development (Likert and visual analog scale) to increase the assessments' reliability. In empirical studies on ESM data [22], visual analog scales had demonstrated the anchor-influenced results, and therefore, different approaches were tested (eg, add a cursor above the anchor line). Interviews with adolescents indicated that Likert scales are more intuitive if they run from high to low on the screen instead of low to high. In December 2019 and February 2020, user experience tests of the Grow It! app were run (test 2d). Adolescents (N=23) played the Grow It! app for 1 week. On the basis of interviews, we improved and extended the content of the app by (1) adding a limited chat function with predesigned stickers to motivate team members, (2) allowing users to choose from 3 challenges a day for 6 weeks, and (3) adding a tour in which game mechanics are explained. Finally, a handbook for errors arose from our technical test and quality assessment, which was performed by the research team (N=10, test 2e).

### Feasibility and Acceptance Test

In May 2020 and in December 2020, at the first 2 peaks of the COVID-19 pandemic, the Grow It! app was launched to assess the game mechanics and user acceptance of the MVP. Owing to government restrictions, all adolescents had to follow social distancing measures (eg, staying at home because schools were closed). Through (social) media, the app was made available to Dutch-speaking adolescents living in the Netherlands, aged 12-25 years, who owned a smartphone. Participants were consecutively enrolled in a Grow It! team after completion of the baseline questionnaire that was linked to the web-based informed consent procedure on a secure website. This way, participants started with the app as soon as possible.

In total, 685 adolescents (sample 1: mean age 16.19, SD 3.11 years; 193/685, 28.2% boys) played the Grow It! app for 6 weeks, and in the second sample, another 1035 adolescents (sample 2: mean age 18.78, SD 3.51 years; 193/1035, 18.64% boys) played the Grow It! app for 3 weeks. A follow-up questionnaire was filled out by 383 and 644 adolescents for samples 1 and 2, respectively (see Table 1 for demographics). In the Grow It! app, participants were given 5 ESM notifications per day and daily challenges. Users who did not show activity in the app (0 or 1 activity in ESM or challenges) were excluded from the sample, because to evaluate the user experience of the Grow It! app we were interested in participants who were involved in playing the app.

A complete overview of all (ESM) instruments and questionnaires is provided in our internet-based codebook [38]. The outcomes of our feasibility and acceptance test can be found in the *Results* section. Statistical analyses are descriptive (means, SDs, and frequencies) and performed using SPSS (version 25; IBM Corp) [39].

**Table 1:** Sample characteristics and demographics.

			Sample 1					Sample 2		
			App engagement (Grow It! activity; N=685)		Follow-up questionnaire (n=383; 55.9% retention)		App engagement (Grow It! activity; N=1035)			Follow-up questionnaire (n=644; 62.2% retention)
Age (years), mean (SD)			16.19 (3.11)		16.26 (3.07)		18.78 (3.51)			18.48 (3.43)
Gender, n (% boys)			193 (28.2)		100 (26)		193 (18.6)			120 (18.7)
**Education** **level^a^, n (%)**										
	Primary school	30 (4.4)		9 (2.3)		9 (0.9)			6 (1)	
	Low	104 (15.2)		54 (14.1)		167 (16.1)			98 (15.2)	
	Medium	152 (22.1)		92 (24.1)		337 (32.6)			201 (31.2)	
	High	399 (58.2)		228 (59.5)		438 (42.3)			293 (45.5)	
	Other	N/A^b^		N/A		84 (8.1)			46 (7.1)	
**Cultural identity, n (%)**										
	Dutch	622 (90.8)		348 (90.7)		1013 (97.9)			631 (98.1)	
	Mixed	57 (8.3)		4 (1.1)		17 (1.6)			11 (1.7)	
	Other	6 (0.9)		31 (8.1)		5 (0.6)			2 (0.3)	

^a^Low: (preparatory school for) technical and vocational training; medium: (preparatory school for) professional education; and high: (preparatory school for) university.

^b^N/A: not applicable.

### End Product of Developmental Process: The Grow It! App

#### User Journey

**Figure 2:**
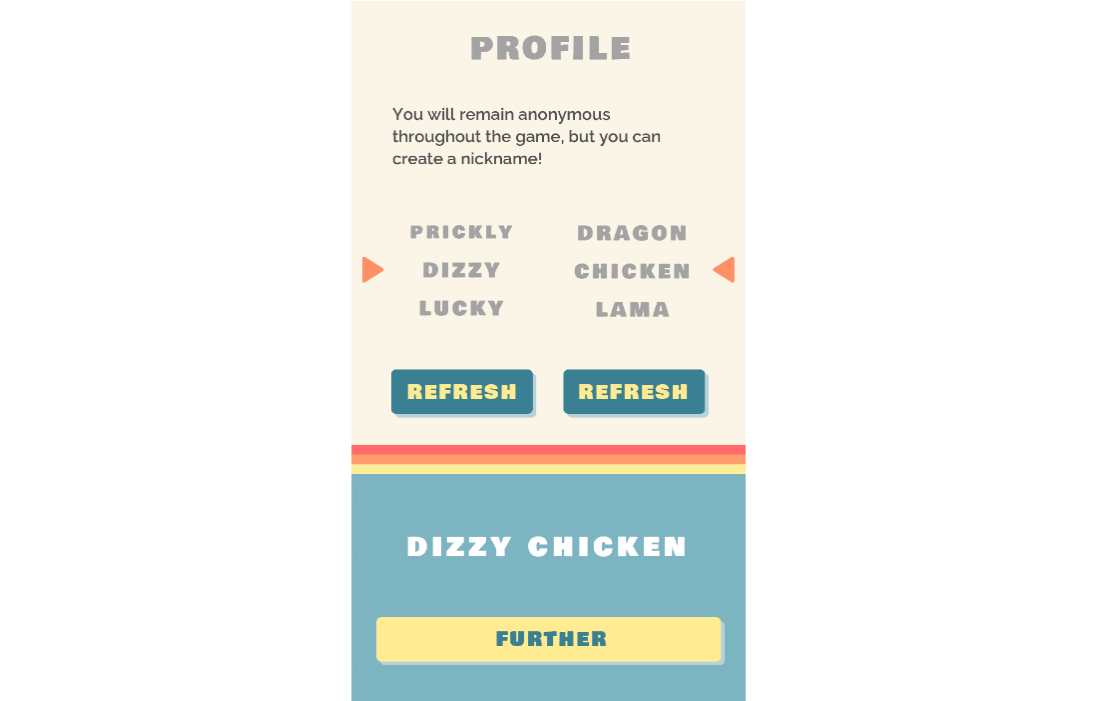
Nickname.

The user journey first entails a phase of enrollment, during which participants can personalize their account. After receiving a 6-digit code (letters and numbers) from the research team via SMS text messaging, they log in to Grow It! and choose their nickname based on 2 turntables. The first turntable shows an adjective (eg, Adorable, Dangerous, Lucky, Creative, and Romantic), and the second turntable shows an animal name (eg, Alpaca, Snake, Iguana, Rabbit, and Crocodile). Participants can rotate the turntables as often as they want to personalize their nickname. For example, one participant nickname could be *Lucky Rabbit* or *Adorable Alpaca* (Figure 2). The game mechanics (ie, personalization, collaboration, competition, and feedback) are explained in the mandatory tour of the Grow It! app.

#### Collaboration

**Figure 3:**
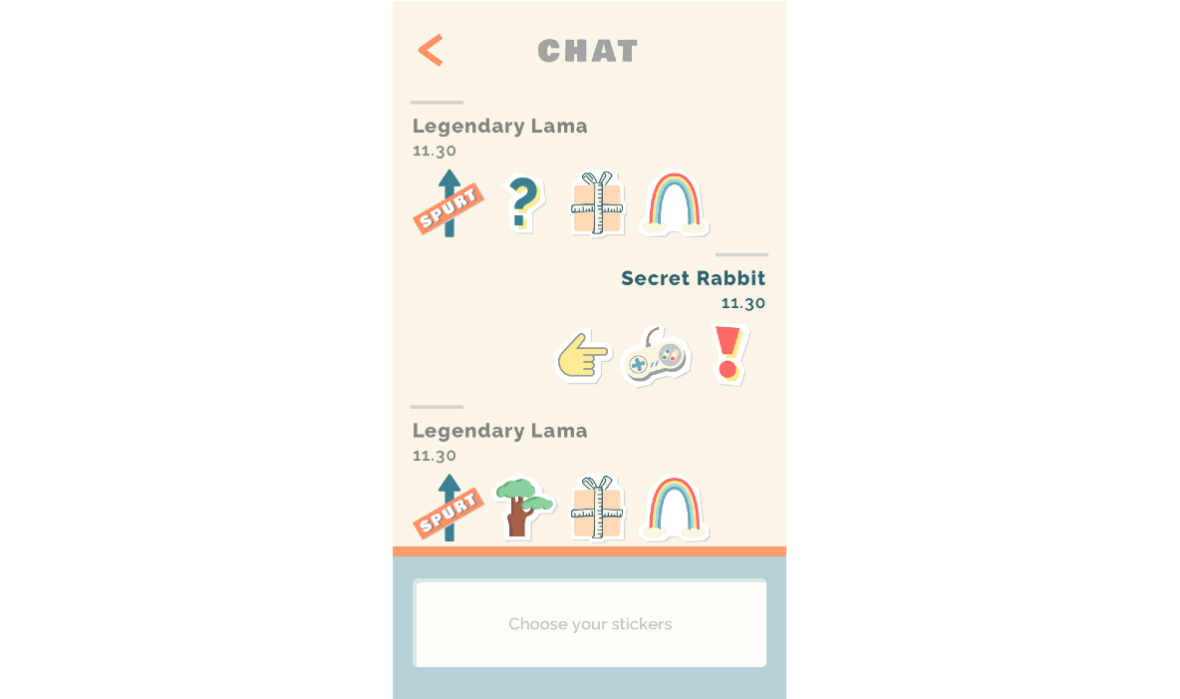
Chat function with positive stickers.

As adolescents are sensitive to peer influence and can be motivated by interactions with peers [40-42], each participant collaborates anonymously in a team with 3 to 7 other players. Adolescents are allocated to a team by the researchers. To support team members, participants can chat by sending and receiving positive stickers (Figure 3). Via this chat system, participants can motivate each other, while the system minimizes the possibilities of bullying and negative peer pressure.

#### Competition

**Figure 4:**
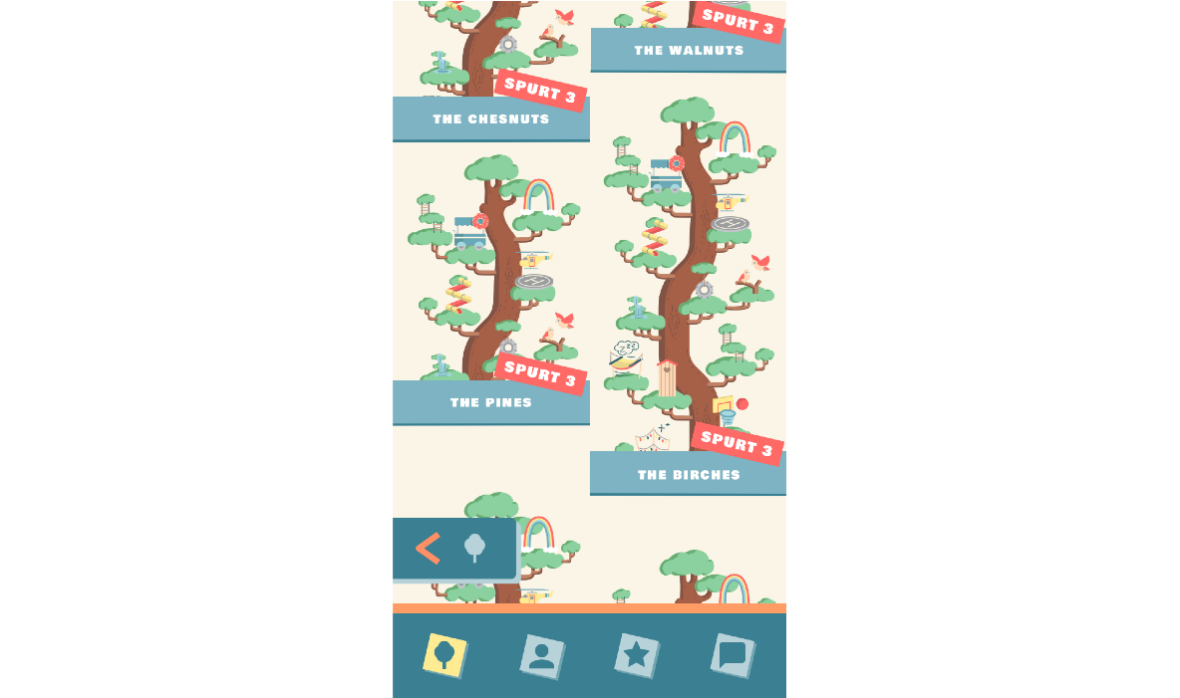
Comparing own tree with trees of other teams.

**Figure 5:**
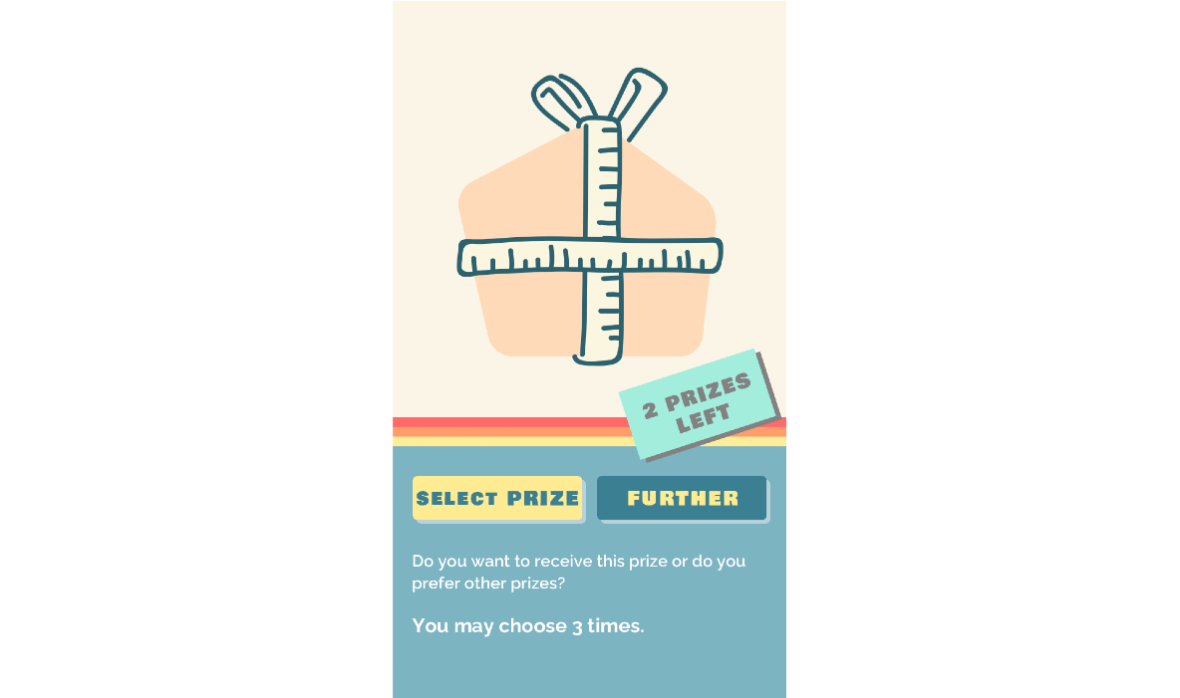
Receiving a gift.

**Figure 6:**
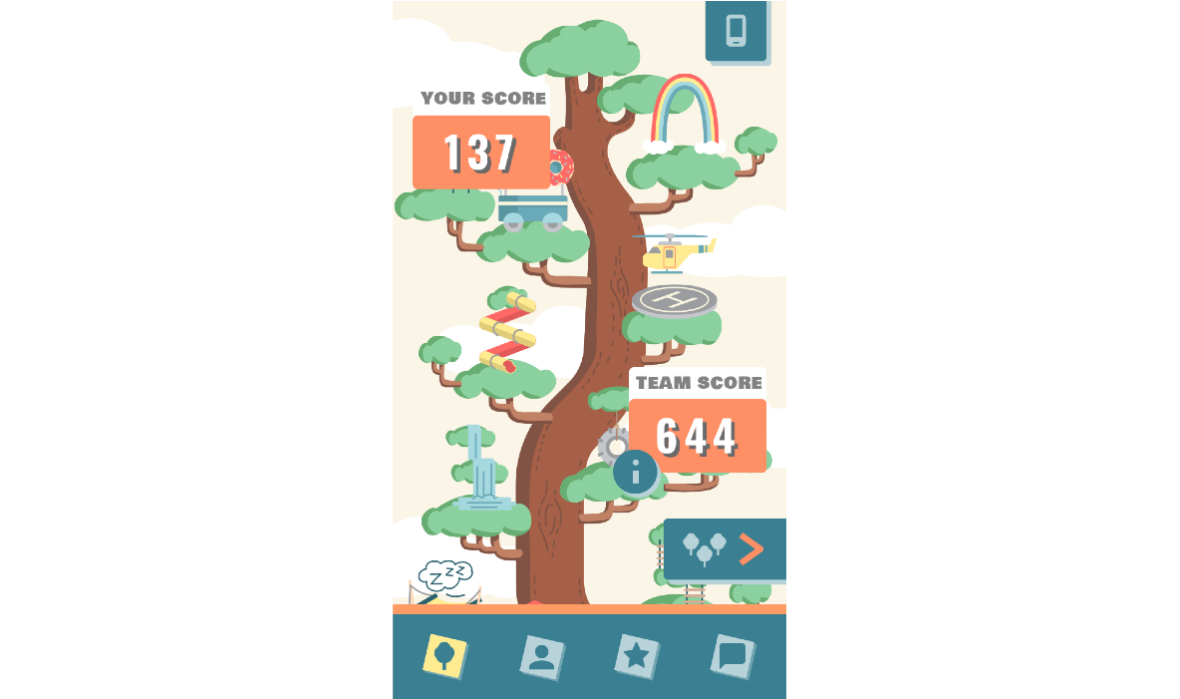
Tree decorated with gifts.

Competition is encouraged at the team level, where teams play versus each other. Each team has a virtual tree with a name (eg, Oaks, Pines, and Palms), which allows participants to compare their team performance with that of other teams (Figure 4). At the start of the game, the tree is empty. Teams *grow* their tree when participants receive points by reporting their feelings and behaviors (ESM) and doing daily challenges. These reports are personal and shielded. Team members only see the amount of points their teammates have collected. After a team collects a specified amount of points, they achieve a *spurt* (ie, level-up), which means that the tree grows in height, and every team member receives a gift to embellish the tree (Figures 5 and 6). As game mechanics, these provide a positive feedback loop and a progress update and establish the reward scheme or the behavioral conditioning that increases retention. Upon earning a gift (ie, loot box) from a growth spurt, a participant can then select his or her choice from 3 gifts. The gifts are wrapped so that there is no indication of what is inside. As a game mechanic, selecting a random gift creates surprise and moments of anticipation essential to maintaining a state of play [43]. When teams have just started using the app, it does not take many points to achieve the first *spurt* and earn a gift. As the game progresses, however, and teams move to higher levels, more and more points are needed to earn a *spurt* and gifts. In this way, adolescents are stimulated to keep playing and remain engaged with the app. The difficulty level is scaffolded by incrementally increasing difficulty, which supports retention by continuously challenging the participants as they progress through the game.

#### Feedback

**Figure 7:**
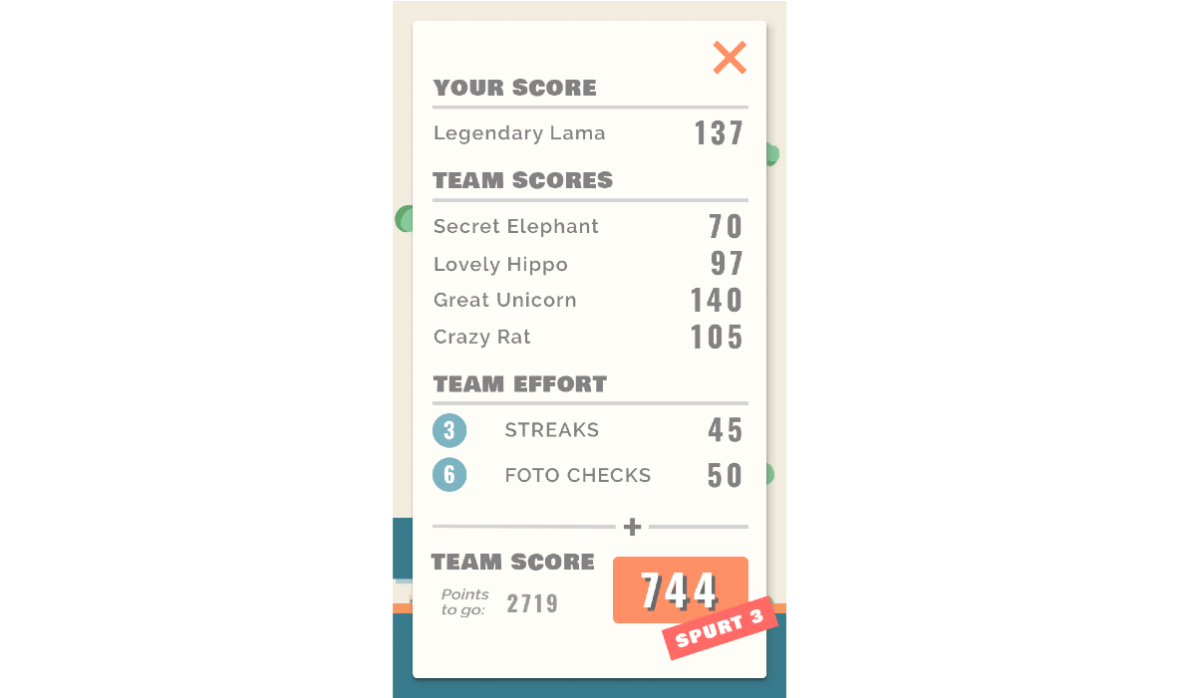
Overview of earned points.

**Figure 8:**
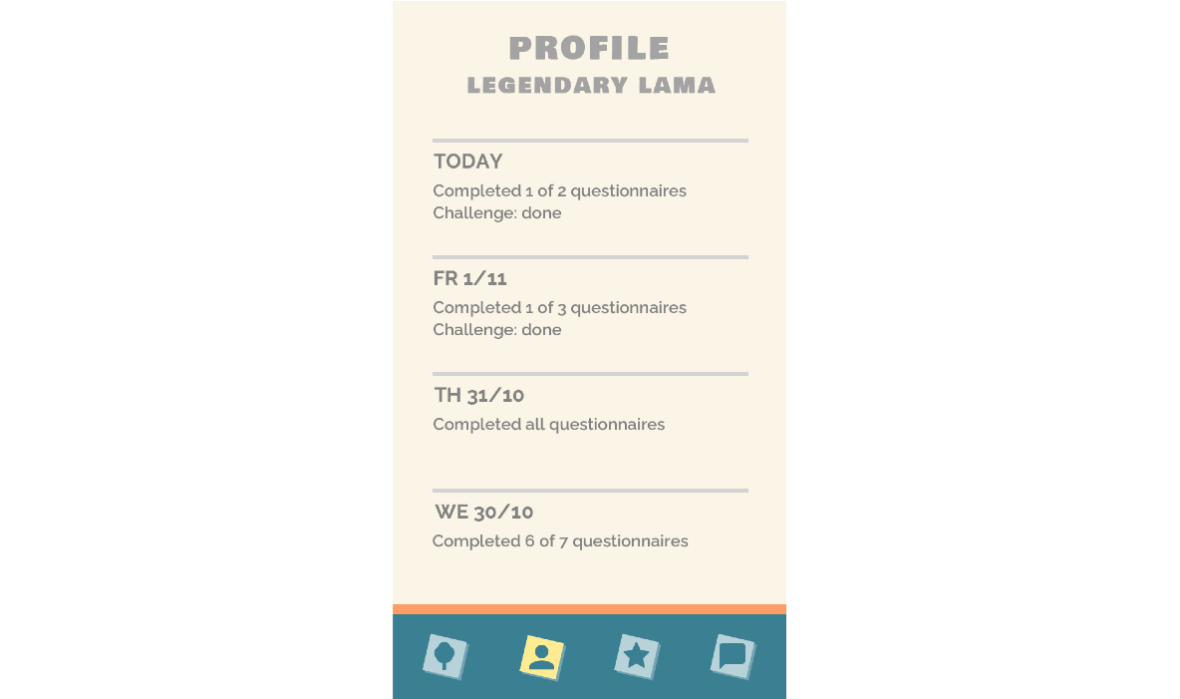
Profile page with the number of completed questionnaires.

The game mechanics of Grow It! provide feedback at different levels. Whereas users can see their own and their team members’ scores in the score overview screen (Figure 7), on their profile page, they obtain an overview of how many times they reported their feelings, behaviors, and challenges that day (Figure 8). Finally, the Grow It! app has a contact button in case of technical issues or urgent psychological problems. Whenever a participant pushes the contact button, a phone number is displayed through which the research team can be reached by telephone or texting on working days during office hours. On the study website, information can be found on how to reach help in acute situations or outside working hours, referring to professional and free services.

#### Daily Emotions

**Figure 9:**
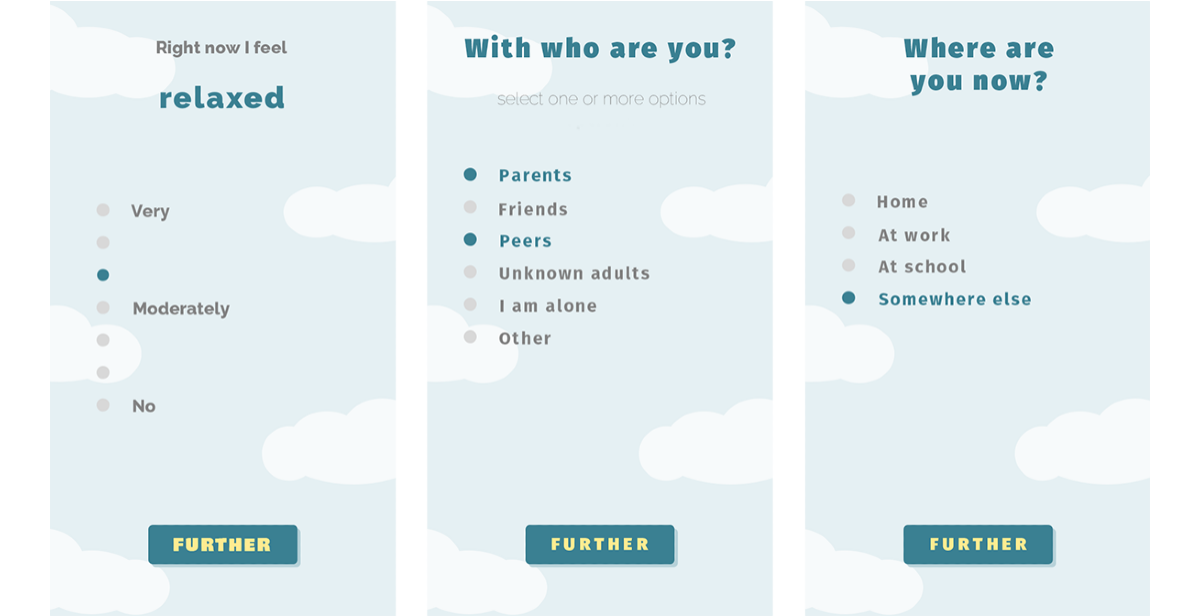
Examples of daily experience sampling method questions.

The ESM is an integral part of the app, which can be used for early identification of emotional problems and enhancing self-management in adolescents [22]. To prompt adolescents to report on their feelings, they receive several notifications per day, which are randomized to prevent structural answering patterns (eg, always in math class at 11 AM) [22]. In the first studies, adolescents answered 5 microquestionnaires per day (taking approximately 1-2 minutes) regarding their sleep, activity, affective well-being (eg, I feel happy or sad), coping strategies, pain, fatigue, social behavior, loneliness, stress, and coping (Figure 9).

Our ESM approach's novelty is that it is gamified to increase motivation, which intends to result in a higher rate of compliance (percentage completed self-evaluations) as well as improved data quality [15]. Compliance is one of the critical quality markers for ESM studies [22].

#### Daily Challenges

**Figure 10:**
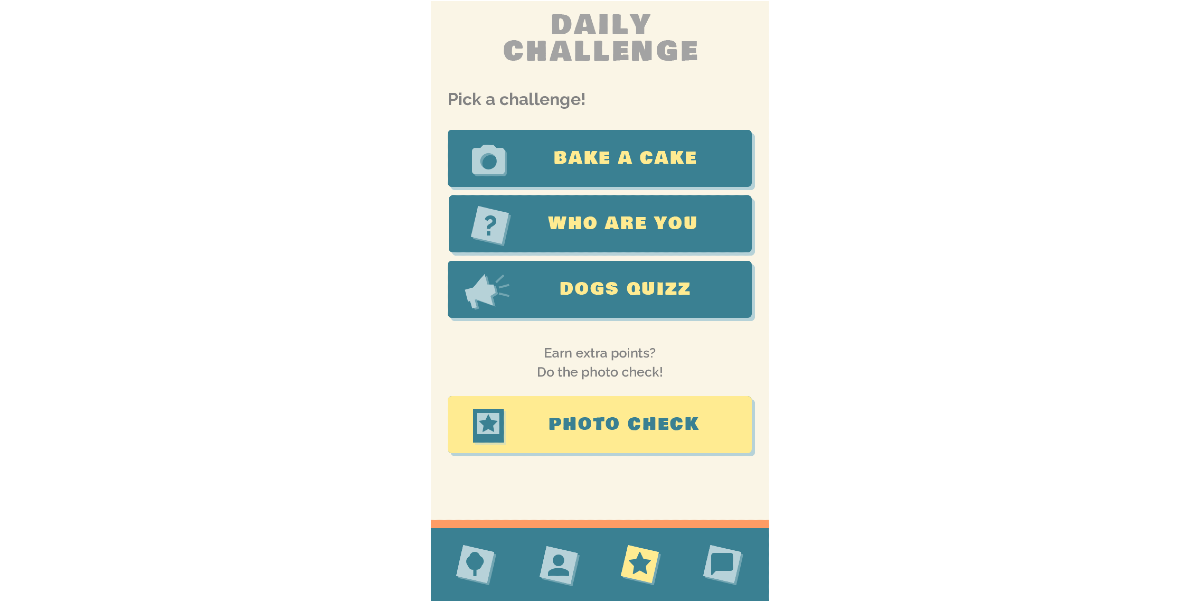
Daily challenges.

To teach adolescents how to cope with setbacks and to promote emotional resilience, the Grow It! app contains daily challenges aimed at strengthening adaptive coping, supporting physical activation, and preventing emotional problems. Coping styles incorporated in the challenges promote distraction, problem solving, social support, and acceptance [44,45]. Participants can choose 1 out of 3 challenges per day (Figure 10). Challenges are divided into three categories: photo challenges, quizzes, and assignments. Examples of challenges are as follows: make a picture of something you hold dear (photo challenge; aimed at distraction), ask someone what they like about you and write it down (assignment; aimed at social support), or answer a multiple-choice question such as *What is sushi usually rolled in?* (quiz; aimed at problem solving). An additional randomly available assignment is the photo check. Participants are shown a matrix of 9 photos and assess which photos fit a particular theme. In this way, they act as the photo challenge jury and can award points to participants and earn extra points themselves for this task.

### Ethical Considerations and Privacy

Risks related to privacy were mitigated by making all participants pseudonymous and only identifiable in the app by participation codes and pseudonyms. Participants determine their pseudonym (ie, nickname) from a preselected set of words provided by the app. Participants cannot be identified, and only the research team has insight into the private data of the participants. Data collected with the app pertain to the user's game data (eg, game-specific actions) and responses to the ESM. The app also accesses the mobile device's camera, but only when a participant takes a photo for a challenge (not at other times), and the app does not access other functionalities (eg, Google, GPS, or health apps). All user data are encrypted and sent directly to a secure server at the researcher’s institute. The privacy and security of the Grow It! app is approved by the privacy and security office of Erasmus Medical Center, and the app complies with the Dutch General Data Protection Regulation (Algemene Verordening Gegevensbescherming) and NEN-norm 7510:2017 (Dutch standard of information security management systems in health care). The app is available for research purposes in the Google Play store [46] and Apple store [47]. This study has been approved by the Medical Ethics Committee of the Erasmus Medical Center (MEC-2020-0287).

## Results

### Overview

As shown in Table 1, participants of sample 1 (N=685; mean age 16.2, SD 3.1 years) were somewhat younger than adolescents in sample 2 (N=1035; mean age 18.8, SD 3.5 years). In general, participants of both samples were relatively highly educated and mostly of Dutch ethnicity. The follow-up questionnaire was filled out by 55.9% (383/685) and 62.2% (644/1035) of the users in sample 1 and sample 2, respectively.

### Feasibility and Acceptance

An overview of app engagement (ESM compliance and challenges) and answers of the user evaluation questionnaire carried out in the follow-up can be found in Tables 2 and 3.

Regarding the ESM component, overall compliance was 16.7% (35.1/210 notifications, sample 1) and 30.9% (32.5/105 notifications, sample 2). About 56.77% (583/1027) of the participants indicated that they thought the number of questions per day was too high, whereas about 40.7% (418/1027) indicated the number of questions as sufficient. Some participants also reported that they did not understand why the same questions were asked repeatedly (sample 1: N=20, sample 2: N=16; ie, at each notification, the same ESM questions were asked to monitor their feelings and behavior over the study weeks). Moreover, 15% (97/644) to 22.9% (88/383) reported no effect of the ESM. However, 66.8% (256/383) to 72.4% (466/644) reported reflecting more on their feelings as a result of the ESM.

With regard to the daily CBT-based challenges, participants completed 31.5% (216/685) to 49.47% (512/1035) of all challenges. Whereas in sample 1, a total of 1.2% (8/685) participants completed all 42 challenges (100%), 6.57% (68/1035) participants completed all 21 challenges (21/21, 100%) in sample 2. The self-reported effect of the challenges showed that 20.6% (79/383) to 44.2% (285/644) of the participants became more active as a result of the Grow It! challenges.

The overall user evaluation of Grow It! was positive. The average app evaluation score was 7.1 of 10 (SD 1.5) in sample 1 and 7.2 of 10 (SD 1.3) in sample 2. Moreover, the app’s design was evaluated with a score of 7.7 to 8.0 of 10. Finally, 72.6% (278/383) to 75.6% (487/644) would recommend the app to their friends.

**Table 2:** App engagement and user evaluation of Grow It! (sample 1).

		Total	Aged 12-17 years	Aged 18-25 years	Difference test (aged 12-17 vs 18-25 years)								
					*t* test (*df*)		Chi-square (*df*)				*P* value		
**App engagement (Grow It! activity)**									N/A^a^				
	Number of users	685	500	185	N/A						N/A		
	Compliance of ESM^b^ (n=210), number of notifications (%)	35.1 (16.7)	34.4 (16.4)	41.8 (19.9)	1.89 (683)						.06		
	Challenges (n=42), n (%)	13.2 (31.5)	13.3 (31.6)	13.1 (31.2)	0.19 (683)						.85		
**User evaluation (follow-up questionnaire)**									N/A				
	Number of users	383	273	110	N/A						N/A		
	Evaluation of the app (1-10), mean (SD)	7.1 (1.5)	7.4 (1.3)	6.6 (1.7)	4.70 (381)						<.001		
	Evaluation of the design (1-10), mean (SD)	7.7 (1.5)	7.8 (1.5)	7.5 (1.5)	2.21 (381)						.03		
**Self-reported effect of ESM, n (%)**						N/A		1.99 (1)				.58	
	I got to know myself better	20 (5.3)	17 (6.3)	3 (2.9)									
	It made me feel better	19 (5)	13 (4.7)	6 (5.7)									
	It made me think about how I feel more	256 (66.8)	181 (66.4)	75 (67.6)									
	No effect	88 (22.9)	62 (22.5)	26 (23.8)									
**Evaluation amount of ESM per day, n (%)**						N/A		9.18 (1)				.002	
	Few	15 (4)	8 (3)	7 (6.5)									
	Sufficient	155 (40.3)	122 (44.8)	32 (29)									
	A lot	213 (55.7)	143 (52.2)	71 (64.5)									
**Self-reported effect of challenges, n (%)**						N/A		5.26 (1)				.26	
	I have had more contact with others	10 (2.5)	6 (2.3)	3 (2.9)									
	I am better at solving problems	8 (2.2)	4 (1.5)	4 (3.8)									
	I am better at accepting situations	32 (8.3)	22 (8.1)	10 (8.7)									
	I have become more active	79 (20.7)	64 (23.6)	15 (13.5)									
	I have started to feel less lonely	28 (7.4)	20 (7.3)	8 (7.7)									
**Evaluation chat function, n (%)**						N/A		5.79 (1)				.22	
	Not nice at all	79 (20.7)	51 (18.7)	28 (25.5)									
	A little bit nice	175 (45.7)	122 (44.7)	53 (48.1)									
	Quite nice	54 (14.1)	41 (14.9)	14 (13.3)									
	Nice	45 (11.7)	33 (12.2)	11 (10.4)									
	Very nice	30 (7.9)	26 (9.5)	4 (3.8)									
Would recommend Grow It! to friends, n (%)		278 (72.6)	212 (77.7)	66 (60)	N/A		12.28 (1)				<.001		
Want to help with future development of the app, n (%)		133 (34.7)	93 (33.9)	41 (36.9)	N/A		0.35 (1)				.55		

^a^N/A: not applicable.

^b^ESM: experience sampling method.

**Table 3:** App engagement and user evaluation of Grow It! (sample 2).

			Total		Aged 12-17 years		Aged 18-25 years		Difference test (aged 12-17 vs 18-25 years)									
									*t* test (*df*)			Chi-square (*df*)				*P* value		
**App engagement (Grow It! activity)**														N/A^a^				
	Number of users	1035		405		630		N/A							N/A			
	Compliance of ESM^b^ (n=105), number of notifications (%)	32.5 (30.9)		30 (28.6)		33.9 (32.3)		2.17 (1033)							.03			
	Challenges (n=21), n (%)	10.4 (49.5)		10.5 (50.1)		10.3 (49.2)		0.45 (1033)							.65			
**User evaluation (follow-up questionnaire)**														N/A				
	Number of users	644		256		388		N/A							N/A			
	Evaluation of the app (1-10), mean (SD)	7.2 (1.3)		7.5 (1.3)		6.9 (1.3)		1.59 (624)							.11			
	Evaluation of the design (1-10), mean (SD)	8.0 (1.3)		8.2 (1.3)		7.8 (1.3)		4.48 (624)							<.001			
**Self-reported effect of ESM, n (%)**										N/A			8.54 (1)				.003	
	I got to know myself better	62 (9.6)		27 (10.4)		35 (9)												
	It made me feel better	19 (3)		13 (5.2)		6 (1.5)												
	It made me think about how I feel more	466 (72.4)		174 (68.0)		292 (75.3)												
	No effect	97 (15)		42 (16.4)		55 (14.2)												
**Evaluation amount of ESM per day, n (%)**										N/A			5.77 (1)				.06	
	Few	11 (1.7)		5 (1.8)		7 (1.7)												
	Sufficient	263 (40.9)		119 (46.5)		144 (37.2)												
	A lot	370 (57.4)		132 (51.6)		237 (61.2)												
**Self-reported effect of challenges, n (%)**										N/A			10.47 (1)				.001	
	I have had more contact with others	106 (16.5)		31 (12)		78 (20)												
	I am better at solving problems	29 (4.5)		16 (6.3)		12 (3)												
	I am better at accepting situations	120 (18.7)		47 (18.3)		74 (19)												
	I have become more active	285 (44.2)		121 (47.2)		162 (41.7)												
	I have started to feel less lonely	104 (16.1)		41 (16.2)		62 (16.1)												
**Evaluation chat function, n (%)**										N/A			28.11 (1)				<.001	
	Not nice at all	142 (22)		35 (13.5)		107 (27.6)												
	A little bit nice	288 (44.7)		109 (42.7)		179 (46)												
	Quite nice	106 (16.4)		54 (21.2)		51 (13.2)												
	Nice	77 (12)		41 (16.1)		36 (9.4)												
	Very nice	31 (4.9)		17 (6.6)		15 (3.8)												
Would recommend Grow It! To friends, n (%)			487 (75.6)		215 (84)		272 (70)		N/A			16.12 (1)				<.001		
Want to help with future development of the app, n (%)			263 (40.9)		117 (45.7)		146 (37.7)		N/A			0.74 (1)				.39		

^a^N/A: not applicable.

^b^ESM: experience sampling method.

### Results for Adolescents Compared With Emerging Adults

Given the broad age range in which adolescents participated (12-25 years), we also reported outcomes separately for adolescents (12-17 years) and emerging adults (18-25 years) in Tables 2 and 3. Higher user evaluations of the Grow It! app were found in the adolescent group in comparison with the emerging adult group (ie, samples 1 and 2: adolescents rated the design of the app higher, and more adolescents would recommend the app to their friends in samples 1 and 2; sample 1: adolescents’ evaluation of the app was higher, and number of ESM notifications per day was evaluated better; sample 2: evaluation of the chat function was better in adolescents). Moreover, no differences between age groups were found with regard to app engagement with the exception of slightly higher compliance of ESM in emerging adults than in adolescents (only in sample 1).

## Discussion

### Principal Findings

The Grow It! app emerged from a lack of and need for preventive interventions for emotional problems and promoting adaptive coping for adolescents; in addition, these interventions need to be low key, nonstigmatizing, fun, attractive, private, and secure. On the basis of the developmental process and acceptance and feasibility of Grow It!, key lessons learned and directions for future research are formulated and shared. Our approach entails an iterative game design process combined with an agile method to develop the smartphone app. The incorporated game features in the Grow It! app were co-designed with adolescent end users to increase participant engagement and adherence. With regard to the app engagement and user evaluation filled out at follow-up, we indicated that we have some evidence that supports the feasibility and acceptance, and therefore applicability, of Grow It! in adolescents.

Earlier studies suggest that adolescents are open to using mHealth [48]. Indeed, the large interest as well as the positive user evaluation provide reason to believe that adolescents, at least a large group, are positive and open to using mHealth. Grow It was co-designed with youths, which was reflected in age-adequate daily challenges and an overall positive rating of the Grow It! app. Moreover, 66.8% (256/383) to 72.4% (466/644) of the participants felt they reflected more on their emotions.

### Limitations

Although the results of the developmental process, acceptance, and feasibility are informative and promising for mHealth, several limitations should be mentioned. First, the majority of the study sample consisted of girls. The design of the app seems more appealing to girls, despite the long process of cocreation with both boys and girls. One of the explanations might be that girls are more inclined to seek help [49] and therefore are also more inclined to participate in our study.

Second, the COVID-19 pandemic and related governmental restrictions might have influenced the results. Owing to remote working during the COVID-19 pandemic, participants had no or minimal personal contact and received minimal instructions on how to participate in an ESM study, although this is stated as an important factor to obtain reliable data [22]. Third, concerning self-monitoring, it is notoriously difficult to motivate adolescents. In this study, compliance of ESM was lower than in a typical research design [22]; however, participants received no financial incentive and were entirely motivated by the game structure to answer questionnaires. We, therefore, expect that with more targeted use (eg, in blended care when the app is explained by a professional), the compliance and user satisfaction might be more favorable. Furthermore, the dropout rates were comparable with other web-based studies [50]. In order to increase app engagement, improvements are needed; for instance, designs may need to be tailored to the individual (personalization) and (in-person) feedback is needed.

### Future Directions

With regard to the early identification of emotional problems, ESM data of the Grow It! app provides an opportunity to develop algorithms in future research for the early detection of emotional problems, which often go unnoticed in adolescents [6]. Identifying emotional problems early on would require capitalizing on novel developments in clinical psychology [51] combined with the motivational game architecture codeveloped with youths [36]. The ESM has already shown to be a reliable method to investigate variation in thoughts, feelings, and symptoms over time and context in research settings [22,23]. Dietvorst et al [25] have demonstrated that ESM data helps to identify the onset of depressive feelings among adolescents 3 months ahead. Specifically, this was done by differentiating typical adolescents (eg, grumpy at home), from early depressive feelings. In future work, analyzing highly rich ESM data with more powerful analytical techniques, such as machine learning, could potentially improve this early identification.

In clinical practice, self-management and self-insight may be enhanced by obtaining insights into one's emotion dynamics [22,26,27]. A feature such as providing participants with feedback through a daily life emotion chart (eg, mood profile) could provide participants with better insight and feedback into their well-being [52,53]. It may also serve as a therapeutic function, as integrating real time mood profiles in the app could encourage adolescents to reflect more on their emotions, coping, and behavior in different contexts [26] and could also be used as a routine outcome measure during therapy. To test the effects of the app upon adolescent well-being and resilience, an additional in-depth evaluation is required. Research questions and hypotheses that are beyond the scope of the developmental process focusing on the main effect of the app are preregistered [54,55] and will be executed in the future accordingly, including a randomized controlled trial study to test the effectiveness of the Grow It! app.

### Conclusions

The Grow It! app has been developed and improved through iterations in collaboration with a large multidisciplinary team. It is innovative, age-attuned, easily accessible, fun, and visually appealing and, most importantly, serves the needs of adolescents. The app was well received by adolescents, and the first findings presented here indicate that adolescents were motivated by the game mechanics of Grow It! and liked completing the ESM questionnaires and daily challenges. Initially, the app was developed for high-risk adolescent populations, such as adolescents with chronic somatic conditions, offspring of parents with psychiatric disorders, or adolescents experiencing extreme stressful societal circumstances, for example, the COVID-19 pandemic. However, the app may also serve a broader purpose of prevention for adolescents from a general population. Our findings support the feasibility and acceptance, and therefore applicability, of the Grow It! app in adolescents.

The ambition is to further improve the app after each research study by including new features and resolving usability issues. The next step will be to focus on the increase of compliance and providing participants feedback through their personal mood profiles.

## Abbreviations

CBT: cognitive behavioral therapy
ESM: experience sampling method
mHealth: mobile health
MVP: minimal viable product
